# Characteristics and Outcomes of Pars Plana Vitrectomy for Proliferative Diabetic Retinopathy Patients in a Limited Resource Tertiary Center over an Eight-Year Period

**DOI:** 10.1155/2019/9481902

**Published:** 2019-03-17

**Authors:** Janejit Choovuthayakorn, Preeyanuch Khunsongkiet, Direk Patikulsila, Nawat Watanachai, Paradee Kunavisarut, Voraporn Chaikitmongkol, Nimitr Ittipunkul

**Affiliations:** Department of Ophthalmology, Faculty of Medicine, Chiang Mai University, Thailand

## Abstract

**Purpose:**

To report characteristics and visual results in patients with PDR-associated complications following pars plana vitrectomy (PPV) in a tertiary center over an eight-year period.

**Methods:**

Medical records of diabetic retinopathy patients receiving PPV between January 2007 and December 2014 for PDR-related complications were reviewed.

**Results:**

A total of 890 consecutive PDR patients were included in the study. PPVs were performed for tractional retinal detachment (36.6%), persistent vitreous hemorrhage (VH) (35.4%), combined tractional and rhegmatogenous retinal detachment (14.5%), and vitreoretinal abnormalities (13.5%), respectively. Preoperative intravitreal bevacizumab injection (IVB) within two weeks prior to PPV and 23G vitrectomy systems was found to decrease the risk for intraoperative retinal break development (*P*=0.045 and *P*=0.015, respectively). The incidence of early dense postoperative VH decreased significantly with the administration of preoperative or intraoperative IVB at the end of PPV. Postoperative visual results significantly correlated with the initial visual acuity level, intraoperative retinal break development, and retained silicone oil tamponade at the final visit (*P* < 0.001, *P*=0.040, and *P*=0.044, respectively). Administration of adjuvant IVB either before or at the end of PPV had no significant association with the final visual outcomes.

**Conclusions:**

This study reported an improvement in visual acuity in nearly half of patients receiving PPV for PDR-associated complications in a limited resource center. The incidence of intraoperative retinal break and early postoperative VH decreased significantly with the application of IVB injections. Poor final visual outcomes were related to the lower initial visual acuity levels, intraoperative retinal breaks, and postoperative retained silicone oil.

## 1. Introduction

Proliferative diabetic retinopathy (PDR) has been a major socioeconomic concern leading to visual impairment and daily life disability despite numerous advancements in diabetic retinopathy screening and treatment [1–4]. Certain PDR-associated conditions including persistent vitreous hemorrhage (VH), tractional retinal detachment (TRD), and combined tractional and rhegmatogenous retinal detachment (combined TRD/RRD) still require vitreoretinal surgery to either maintain or improve the vision [5]. In recent years, the introduction of intravitreal antivascular endothelial growth factor (anti-VEGF) injection has been demonstrated, although inconsistent, to be a factor in dramatic improvements in PDR-receiving PPV patients. This study aimed at evaluating characteristics and visual and anatomical outcomes for PDR patients who underwent PPV over an eight-year period in a tertiary care referral center with limited resources. The results provide information for patients in making a decision and for physicians counselling PDR patients who require surgical intervention.

## 2. Methods

This observational study was approved by the Chiang Mai University Hospital Research Ethics Committee. The protocol was performed according to the tenets of the Declaration of Helsinki. The medical records of consecutive PDR patients undergoing primary PPV due to PDR-associated conditions between January 2007 and December 2014 were retrospectively reviewed. Demographic data, ophthalmologic findings, surgical procedures, and visual and anatomical outcomes were collected. Indications for surgery were categorized into four groups: persistent or recurrent VH; foveal threatening or foveal involved TRD; combined TRD and RRD; and vitreomacular interface abnormalities (VMI) including excessive macular traction. If both eyes were eligible, the eye with the worse initial VA was included. An adjuvant of intravitreal bevacizumab (IVB) injection before or at the end of primary PPV was noted. The injection within four weeks prior to PPV was defined as a preoperative procedure. Vitrectomy systems, either 20G or 23G, were of vitreoretinal specialists' preferences. The presence of postoperative VH was assessed in patients who did not attain primary silicone oil tamponade and was followed for at least four weeks. Postoperative VH occurring within four weeks following primary vitrectomy was considered as the early onset, while VH occurring after that period was defined as the late onset. Postoperative VH grade 3 or more in which fundus details could not be evaluated, but optic disc could be identified was referred to as dense VH. Final visual and anatomical outcomes were analyzed in patients who were followed for three months or more. Poor visual outcome was determined as a visual acuity of 3/60 or worse. Anatomical success was defined as the flattening of the retina within the major vascular arcade.

### 2.1. Statistical Analysis

Descriptive analysis was used to present patients' demographic data. VA was converted from the Snellen to logMAR (logarithm of the minimum angle of resolution) unit for statistical analysis. Continuous data were evaluated with ANOVA or Kruskal Wallis when appropriate. Categorical data were evaluated with chi-square or Fisher's exact test. After adjusting for age, gender, initial VA, vitrectomy system, timing of IVB, surgical indication, and combined cataract and PPV procedure, factors associated with intraoperative retinal breaks, postoperative early dense VH development, and final visual outcomes were considered using logistic regression analysis. A *P* value of less than 0.05 was considered significant.

## 3. Results

Eight hundred and ninety medical records of consecutive PDR patients receiving PPV for diabetic retinopathy complications: 326 (36.6%) patients for TRD, 315 (35.4%) for VH, 129 (14.5%) for combined TRD and RRD, and 120 (13.5%) for VMI abnormalities, were identified and reviewed. Overall, the mean age (SD) of patients was 51.9 (10.1) years. The proportion of females: 536 (60.2%) patients, was slightly higher than males. Initial phakic lens status was presented in 817 (91.8%) patients. In the initial phakic eyes, primary PPV combined with cataract extraction was performed in 211/817 (25.8%) eyes, while subsequent cataract surgery following primary PPV was performed in 203/817 (24.8%) eyes. During primary vitrectomy, endolaser photocoagulation was performed in 833 (93.6%) eyes, primarily due to the absence of preoperative panretinal photocoagulation (PRP) (*N*=360, 43.2%) and the incomplete preoperative PRP (*N*=473, 56.8%).  Table 1 summarizes patients' characteristics and perioperative procedures by surgical indication.

### 3.1. Intraoperative Complications

Intraoperative lens injury occurred in 4 (0.4%) eyes. Intraoperative retinal break occurred in 67 (7.5%) eyes during primary PPV, 47/456 (10.3%) eyes among the 20G group, and 20/434 (4.6%) eyes of the 23G group. Multivariate analysis showed that the TRD group (*P*=0.006; odds ratio, 5.374; 95% confidence interval, 1.615–17.885) and the combined TRD/RRD group (*P*=0.014; odds ratio, 5.003; 95% confidence interval, 1.393–17.967) had higher associations with the development of intraoperative retinal breaks, while the VH group showed no difference in risk when compared to the VMI group. In addition, preoperative IVB administered within two weeks prior to PPV (*P*=0.045, odds ratio, 0.487; 95% confidence interval, 0.170–0.987) and 23G PPV system (*P*=0.015; odds ratio, 0.500; 95% confidence interval, 0.286–0.874), compared to 20G, decreased the risk for the intraoperative retinal break.

### 3.2. Postoperative Vitreous Hemorrhage

Six hundred and seventy-three patients who did not receive silicone oil tamponade at the end of primary PPV and were followed for one month or more were noted. Of those, early dense postoperative VH developed in 114 (16.9%) eyes in which 70 (10.4%) required further intervention to clear the vitreous cavity. Multivariate regression for early dense postoperative VH is shown in Table 2. Late postoperative VH occurred in 37 (5.5%) eyes. Preoperative IVB and/or at the end of PPV did not reveal a significant association with the incidence of the late onset postoperative VH (*P*=0.91).

### 3.3. Final Outcomes

Among 792 patients who were followed for three months or more, postoperative RD following primary PPV developed in 46 (5.8%) eyes (two in the VMI group, 41 in the TRD and combined TRD/RRD group, and three in the VH group, respectively). Eighty-eight eyes retained silicone oil tamponade at the last follow-up (two in the VMI group, 77 in the TRD and combined TRD/RRD group, and nine in the VH group, respectively). Final flattening of the posterior retina was observed in 769 (97.1%) eyes, including 72 (9.1%) eyes that were flattened under silicone oil tamponade. Final visual status compared to the initial preoperative VA separated by surgical indications was analyzed (Table 3).  Table 4 shows factors related to poor final visual outcomes by multivariate analysis. Administration of adjuvant IVB either before or at the end of PPV had no significant association with visual outcomes. As far as other postoperative complications, 58 (7.2%) eyes developed glaucoma requiring medical treatment and 33 (4.2%) eyes developed neovascular glaucoma (NVG). Patients who underwent only PPV had no significant difference in risk for postoperative NVG development compared to patients who underwent combined PPV and cataract surgery (24 versus 9 patients, *P*=0.42). None developed suprachoroidal hemorrhage nor endophthalmitis.

## 4. Discussion

Regarding PPV for PDR-associated with diabetic retinopathy complications, this study explored that nearly half of the patients achieved visual improvement. Use of a 23G vitrectomy system and the administration of IVB prior to PPV were associated with a lower incidence of intraoperative retinal breaks. Poor initial VA level, the presence of intraoperative retinal break, and the silicone tamponade status at the final visit were associated with poor final visual outcome.

Complications attributed to vitrectomy in PDR patients, causing either temporary or eventually permanent postoperative visual impairment, have been reported in a number of previous studies [6–9]. However, more favorable outcomes following PPV have been reported, implying a microincisional vitrectomy system and administration of intravitreal anti-VEGF injection as a surgical adjuvant [10–13]. Publications regarding performing 20G PPV for PDR complications without an adjunct of the intravitreal pharmacologic agent variously described an occurrence of intraoperative retinal breaks from 14% to 42%. With a limited number of eyes studied, Oshima et al. and Park et al. did not detect a difference in intraoperative retinal break development compared between the 23G and the 20G PPV systems [14, 15]. This study, in accordance with others, demonstrated a lower rate of intraoperative retinal break formation in the 23G PPV group compared to the 20G group [16, 17]. Intraoperative retinal tear occurrence was proposed as a risk factor for postoperative retinal detachment, necessitating additional intervention following PPV in PDR cases [7]. Then, a surgical approach that minimizes the incidence of intraoperative retinal breaks is a preferable surgical management. Several designs of microincisional vitrectomy systems have recently gained widespread application. With limited to 23G systems in this setting, the benefit for lowering the chance of intraoperative retinal breaks has been observed. For the role of IVB, several reports showed the efficacy of preoperative IVB with the feasibility of PPV. However, various pretreatment timings, ranging from one to three days, up to four weeks prior to PPV, and various doses, ranging from 1 mg to 2.5 mg, were given [14, 18–22]. This study observed a protective effect of 1.25 mg IVB administered within two weeks before PPV in decreasing retinal breaks, while a longer pretreatment interval did not show any relevant benefit.

Regarding postoperative VH, inconsistent evidence of either preoperative IVB or at the end of PPV to prevent POVH has been published [19–26]. PDR patients who underwent PPV without an IVB adjuvant, an incidence of postoperative VH, have been reported from 17% to 60%, while an incidence of 11% to 38% was shown in the group who received IVB [8, 22, 25–27]. Göncü et al. did not demonstrate an efficacy of IVB at the end of PPV for nonclearing VH groups [26]. On the contrary, Cheema et al. found that nonclearing VH and TRD patients receiving IVB at the end of surgery had a significantly less risk for an episode of recurrent dense VH after the first four weeks following PPV [28]. However, a difference in inclusion criteria, severity of the vascular component, and definition of postoperative VH may preclude comparison between reports. VH patients were less likely to have a reopening of fibrovascular tissue remnant or rebleeding from injured retinal vessels during surgery compared to TRD or combined TRD/RRD patients. This study showed that patients with combined TRD/RRD, pure TRD, and VH had a higher risk of early postoperative VH compared to VMI patients. In addition, pretreatment two weeks prior to PPV and/or IVB injection at the end of surgery was associated with a reduction of early dense postoperative VH.

In view of postoperative VA at the final follow-up, although VA was not improved in all patients after diabetic vitrectomy, nearly half of patients in the VMI and TRD groups and more than half of patients with VH in this study gained two or more lines of vision. Less than one-fifth of the patients in this study sustained visual deterioration of two lines or more, comparable to a range of 11 to 27% in other publications [9, 29]. However, a discrepancy in the literature still exists regarding IVB injection and postoperative functional outcomes. According to evidence from meta-analysis, Smith et al. reported no significant effect of IVB either pre- or intraoperative on final VA. Zhoa et al. showed that patients pretreated with anti-VEGF injection achieved significantly better final postoperative BCVA than those who did not [30, 31]. Our study did not find any association between final visual outcomes and intravitreal anti-VEGF injections. The various pathophysiologic factors including preoperative macular status and controlled-associated systemic conditions may explain the variation in visual outcomes. On the contrary, our study showed that the initial VA level, the development of intraoperative retinal breaks, and the retained silicone oil tamponade at the final visit were significantly related to the final VA.

A bias of this study remained from the retrospective nature. First, a grading of the extent of presenting tractional detachment and an exacerbation of fibrovascular membrane contraction following preoperative IVB leading to TRD progression was not evaluated. Second, a location of intraoperative retinal break was not well documented. Third, the bias of physicians' decisions to administer IVB to more severe TRD and VH patients could not be ruled out. Further study to clarify the effectiveness of intravitreal anti-VEGF and PPV for PDR may provide more thorough information.

## 5. Conclusion

Regarding vitrectomy for PDR-associated complications, administering IVB either prior to or at the end of PPV showed a promising effect for a reduction of early postoperative VH although beneficial effects for postoperative visual outcomes could not be achieved. Long-term functional visual results were associated with the initial visual acuity levels and the occurrence of intraoperative retinal breaks.

## Figures and Tables

**Table 1:** Baseline demographics and perioperative procedures for proliferative diabetic retinopathy patients undergoing vitrectomy by surgical indication.

	VMI abnormalities (*N*=120)	TRD (*N*=326)	Combined TRD and RRD (*N*=129)	VH (*N*=315)	*P* value
Mean age (SD), year	56.9 (8.9)	49.3 (9.8)	51.1 (9.5)	52.9 (10.3)	<0.001
Gender, male/female	52/68	116/210	41/88	145/170	0.009
Type II diabetes, *N* (%)	116 (96.7)	306 (93.9)	122 (94.6)	300 (95.2)	0.699
Baseline VA (SD), logMAR unit	1.0 (0.4)	1.6 (0.6)	1.6 (0.7)	1.7 (0.7)	<0.001
23G PPV, *N* (%)	69 (57.5)	139 (42.6)	70 (54.3)	156 (49.5)	0.017
Combined primary PPV and cataract surgery, *N* (%)	42 (35.0)	67 (20.6)	23 (17.8)	79 (25.1)	0.006
Preoperative intravitreal bevacizumab injection within 4 weeks prior to and/or at the end of PPV, *N* (%)	54 (45.0)	180 (55.2)	59 (45.7)	186 (59.0)	0.122
Primary intraoperative silicone oil tamponade, *N* (%)	0 (0)	109 (33.4)	75 (58.1)	7 (2.2)	<0.001

VMI = vitreomacular interface abnormalities; VH = vitreous hemorrhage; TRD = tractional retinal detachment; RRD = rhegmatogenous retinal detachment; SD = standard deviation; VA = visual acuity; VEGF = vascular endothelial growth factor; PPV = pars plana vitrectomy.

**Table 2:** Multivariate analysis for factors associated with postoperative early dense vitreous hemorrhage in proliferative diabetic retinopathy patients who underwent vitrectomy.

	*P* value	Odds ratio	95% CI
Indications for PPV			
** **VMI	—		
** **TRD group	0.003	2.941	1.433–6.036
** **Combined TRD and RRD group	0.042	2.637	1.037–6.708
** **VH group	0.044	2.080	1.020–4.244
Intravitreal bevacizumab injection			
** **2 weeks prior to PPV	0.003	0.167	0.050–0.553
** **At the end of PPV	<0.001	0.219	0.126–0.382
** **Combined 2 weeks prior to/at the end of PPV	0.028	0.391	0.169–0.902

CI = confidence interval; VMI = vitreomacular interface abnormalities; TRD = tractional retinal detachment; RRD = rhegmatogenous retinal detachment; VH = vitreous hemorrhage; SD = standard deviation; PPV = pars plana vitrectomy.

**Table 3:** Postoperative final visual acuity changes for proliferative diabetic retinopathy patients who underwent vitrectomy.

	VMI abnormalities (*N*=113)	TRD (*N*=290)	Combined TRD and RRD (*N*=118)	VH (*N*=271)
FVA 20/40 or better, *N* (%)	34 (30.2)	35 (12.1)	15 (12.7)	98 (36.2)
FVA worse than 20/200, *N* (%)	18 (15.9)	112 (38.6)	45 (38.1)	35 (12.9)
Two lines or more FVA improvement, *N* (%)	51 (45.1)	125 (43.1)	54 (45.8)	204 (75.3)
Two lines or more FVA worsening, *N* (%)	10 (8.8)	46 (15.9)	17 (14.4)	17 (6.3)

FVA = final visual acuity; VMI = vitreomacular interface abnormalities; VH = vitreous hemorrhage; TRD = tractional retinal detachment; RRD = rhegmatogenous retinal detachment.

**Table 4:** Multivariate analysis for factors associated with poor final visual outcome in proliferative diabetic retinopathy patients who underwent vitrectomy.

	*P* value	Odds ratio	95% CI
Indications for PPV			
** **VMI	—		
** **TRD group	0.445	1.279	0.680–2.406
** **Combined TRD and RRD group	0.607	1.208	0.589–2.478
** **VH group	0.003	0.357	0.180–0.710
Initial VA 3/60 or worse	<0.001	5.518	3.490–8.727
Presence of intraoperative retinal break	0.014	2.260	1.178–4.337
Retained silicone oil at the final follow-up	0.044	2.140	1.104–6.608

CI = confidence interval; VMI = vitreomacular interface abnormalities; TRD = tractional retinal detachment; RRD = rhegmatogenous retinal detachment; VH = vitreous hemorrhage; SD = standard deviation; PPV = pars plana vitrectomy.

## Data Availability

The datasets used for this study are available from the corresponding author on reasonable request.
